# Optimum storage conditions for osteochondral allograft plugs: An ex vivo comparative study of 12 storage protocols

**DOI:** 10.1002/jeo2.70206

**Published:** 2025-03-31

**Authors:** Ramin Shayan‐Moghadam, Arash Sherafatvaziri, Fardis Vosoughi, Alireza Mirzamohamadi, Hiva Saffar, Mahdi Shafieian, Leila Oryadi Zanjani, Hossein Nematian, Mohammad Hossein Nabian

**Affiliations:** ^1^ Center for Orthopedic Trans‐Disciplinary Applied Research Tehran University of Medical Sciences Tehran Iran; ^2^ Department of Orthopedic Surgery, Shariati Hospital and School of Medicine Tehran University of Medical Sciences Tehran Iran; ^3^ Department of Pathology, Shariati Hospital Tehran University of Medical Sciences Tehran Iran; ^4^ Department of Biomedical Engineering Amirkabir University of Technology (Tehran Polytechnic) Tehran Iran

**Keywords:** biomechanical properties, cartilage preservation, chondrocyte viability, culture media, dynamic culture conditions, ex vivo study, histopathological analysis, osteochondral allograft, storage protocols, temperature effects

## Abstract

**Objective:**

Physiological storage temperature and chondrogenic supplements can enhance tissue viability, potentially overcoming the limitations associated with osteochondral allograft transplantation. This study aimed to evaluate the quality of macroscopically healthy cartilage across 12 different storage protocols to find optimum storage conditions for osteochondral allograft plugs.

**Methods:**

Osteochondral plugs were obtained from arthroplasty candidates and divided into 12 groups based on two culture media (Media 1 [supplemented Dulbecco's modified Eagle's medium {DMEM}/Ham's Nutrient Mixture F12] or Media 2 [enriched Media 1 with 10 ng/mL of transforming growth factor‐beta {TGF‐β}]), two culture conditions (static or dynamic), and three temperatures (−70°C, 4°C and 37°C). Subsequently, samples were evaluated on Days 1, 14, 28 and 60 for biochemical, biomechanical and histopathological characteristics alongside bacterial surveillance.

**Results:**

A total of 4338 plugs from 843 donors were assessed. Chondrocyte viability and proteoglycan synthesis were highest in the DMEM enriched with TGF‐β at 37°C and 4°C. Although biomechanical properties decreased over time in all groups, dynamic culture conditions resulted in smaller decreases compared to other storage protocols. Viscoelasticity was observed in all samples, with dynamic media groups being maintained the most. Histological evaluation showed signs of degeneration, and temperature variations affected the preservation of the tissue differently. Bacterial surveillance identified contamination in specific storage conditions.

**Conclusion:**

Storing osteochondral allografts at 37°C in TGF‐β supplemented media under dynamic conditions may extend the grafting window from 14 to 60 days. This extension could improve tissue availability, reduce costs and minimize graft wastage, thereby advancing joint resurfacing techniques. Further research is needed to confirm the safety and efficacy of this storage protocol.

**Level of Evidence:**

N/A.

**Abbreviations**
DMEM: Dulbecco's modified Eagle's medium
ECM: extracellular matrix
FGF: fibroblast growth factor
GAG: glycosaminoglycan
HRP: horseradish peroxidase
IGF‐1: insulin‐like growth factor
OCA: osteochondral allograft
OD: optical density
TGF‐β: transforming growth factor‐beta
TKA: total knee arthroplasty

## INTRODUCTION

The limitations of total joint replacements for young active individuals have stimulated the search for alternative treatments in the field of biological joint resurfacing. Multiple alternatives have been described for the treatment of cartilage injuries, including subchondral drilling, microfracture, autologous and allogeneic osteochondral grafts and autologous chondrocyte implantation [18, 23, 29, 30]. When one of the first‐line treatments fails in a young patient, using a fresh osteochondral allograft (OCA) is an alternative to consider, particularly in those secondary to fractures and osteochondritis dissecans [14]. Fresh OCA resurfacing allows the implantation of anatomically matched, mature articular cartilage with viable chondrocytes while avoiding donor‐site morbidity. Furthermore, this technique allows transplantation of both cartilage and underlying bone, enabling surgeons to address pathology extending beyond the subchondral plate [1].

Fresh OCA transplantation involves transplanting mature hyaline cartilage with viable chondrocytes to the lesion site, utilizing grafts that can withstand storage under hypothermic conditions. However, disadvantages include limited donor tissue availability, storage limitations, short time frames for use, and the risk of infectious disease transmission [14, 31]. Traditionally, the procedure has been limited to areas with tissue banks equipped to procure and process tissue. The intact osteochondral specimen is kept at 4°C in either normal saline or lactated Ringer's solution, size‐matched to the recipient, and then transplanted within days of procurement to ensure high chondrocyte viability. Researchers have found that freezing intact articular cartilage, even with cryoprotective agents like dimethyl sulfoxide or with controlled rate freezing techniques, results in either complete or nearly complete loss of chondrocyte viability [20, 22, 36]. Furthermore, current cartilage banking and testing protocols, recommended by the American Association of Tissue Banks, require a 14‐day culturing and disease screening period before releasing grafts for clinical use [9].

Several studies have evaluated the storage conditions of osteochondral allografts to extend the storage as long as possible while maintaining optimal cell viability [3, 17, 35, 39]. It has been shown that, over time, cartilage allograft viability decreases in storage [18]. Using the standard tissue bank practice of 4°C storage, chondrocyte viability deteriorates significantly within the initial 14‐day tissue clearance period [9, 13, 27, 38]. Since chondrocyte viability influences the long‐term clinical success of OCA [8], optimizing storage conditions during the early period after harvesting is crucial. Proposed preservation goals suggest maintaining a minimum viable chondrocyte density of 70% living chondrocytes [8, 33]. Key variables of interest include the type of preservation solution [38, 40, 41], choice of supplementation [5, 28, 35], temperature [4, 27, 41] and level of oxygenation [27]. Although conditions have been assessed across several animal species, there are very few human studies [34]. While both refrigerated conditions at 4°C and physiologic storage at 37°C have shown promise in animal models, comparisons between different temperature conditions in humans are lacking. Therefore, validation in human models is necessary, as variables such as species‐specific core temperature and cartilage morphology may differ.

Transforming growth factor‐beta (TGF‐β), insulin‐like growth factor (IGF‐1), and fibroblast growth factor (FGF) are the most studied growth factors, playing important role in regulating chondrocyte activity and extracellular matrix (ECM) synthesis.

TGF‐β activates the Smad‐dependent pathway to stimulate transcription factors like Sox9, which are essential for chondrogenesis and the reduction of ECM degradation. This pathway also protects the survival of chondrocytes. Moreover, TGF‐β can mediate cartilage homoeostasis by inducing the MAPK pathway [7]. Studies have shown that TGF‐β preserves cartilage integrity by reducing catabolic activity, including the actions of cytokines such as Interleukin‐1 and matrix metalloproteinases [12, 32].

IGF‐1 primarily stimulates chondrocyte growth and proteoglycan synthesis through the PI3K/Akt pathway but is less effective over prolonged exposure. Extended exposure can lead to chondrocyte senescence, indicated by increased p53 and p21 expression and a higher number of SA‐β‐gal positive cells, leading to reduced cell viability and proliferation [43]. This makes IGF‐1 less suitable for long‐term cartilage preservation. In ageing or osteoarthritic cartilage, the reduction of chondrocyte matrix catabolism in response to IGF‐1 may be diminished, making it less suitable for the current study [12]. On the other hand, a previous study has shown that TGF‐β1 compared to IGF‐1 has a more significant influence on the re‐differentiation and proliferation of chondrocytes as well as ECM synthesis (collagen type II and aggrecan) under normoxic conditions [16].

FGF, especially FGF‐2 and FGF‐18, also stimulates chondrocyte proliferation and plays a critical role in cartilage homoeostasis through the RAS/MAPK pathway. However, in osteoarthritic conditions, altered FGF signalling may promote cartilage degradation [6, 42].

While FGF‐2 supports cartilage repair by attracting mesenchymal stem cells to damaged areas, high doses can inhibit proteoglycan synthesis, limiting its utility. FGF‐18 supports chondrocyte proliferation and matrix synthesis, particularly in injured joints [12, 32].

Considering limitations such as cost, the risks associated with prolonged exposure to growth factors, and the characteristics of our samples (sourced from osteoarthritic joints and affected by osteoarthritic conditions despite extensive efforts to ensure high‐quality shiny cartilages), TGF‐β was chosen as the chondrogenic growth factor for investigation in the present study.

This study aimed to evaluate the quality of macroscopically healthy cartilage from middle‐aged patients undergoing total knee arthroplasty (TKA) across 12 different storage protocols to identify optimal conditions for osteochondral allograft plugs. Chondrocyte viability, cartilage histology, biochemical and biomechanical properties of the ECM, and bacterial contamination were assessed after 14, 28 and 60 days of storage. The hypothesis was that enriching the culture media with TGF‐β and utilizing dynamic culture conditions would improve osteochondral allograft preservation compared to standard culture media.

## MATERIALS AND METHODS

This research received approval from the institutional ethics committee on 2022‐02‐12 with the approval code IR.TUMS.MEDICINE.REC.1400.1338. All procedures were performed in compliance with relevant laws, institutional guidelines, and the ethical principles outlined in the Declaration of Helsinki [2].

### Donor selection

The inclusion criteria for donors encompassed osteoarthritis patients aged between 45 and 65 years, who were not suitable candidates for unicompartmental knee arthroplasty due to other reasons (such as posterior erosion, flexion contracture, or patellofemoral distraction) and underwent TKA in three tertiary medical centres affiliated with Tehran University of Medical Sciences from April 2022 to April 2023. All included donors had varus knees and exhibited cartilage destruction in the medial compartment but with relatively intact lateral compartment cartilage. Their lateral femoral condyles had shiny, well‐preserved cartilage with a thickness of more than 6 mm. Osteochondral samples were harvested from the relatively undisturbed zones of the lateral femoral condyle. In some cases, both knees of a patient were operated on (at different times), and samples were collected from the condyles of both limbs. Only articular cartilage that attained a grade of 1A or higher based on the grading system introduced by Noyes and Stabler [25] was considered suitable for inclusion in the ongoing research. On the other hand, those with a background of inflammatory arthritis, malignancies, autoimmune disorders and infection were excluded. Informed consent was obtained from all donors.

### Osteochondral tissue harvesting

Osteochondral tissue specimens were obtained from femoral bone cuts during TKA. Fresh femoral condyles were harvested, and four to six osteochondral plugs were extracted per condyle using a coring drill. Plugs were collected from predefined anatomical sites to standardize and control for regional variations in cartilage properties. Immediately after harvesting, tissue specimens were placed in sterile containers filled with lactated Ringer's solution.

### Transfer and preservation conditions

Within a span of less than 6 h, the samples were conveyed to the laboratory at a temperature of 4°C for subsequent processing. Upon arrival of the samples, the cartilage tissue was assessed by two knee surgeons in terms of the grading system introduced by Noyes and Stabler [25] to confirm suitability for the study.

**Figure 1:**
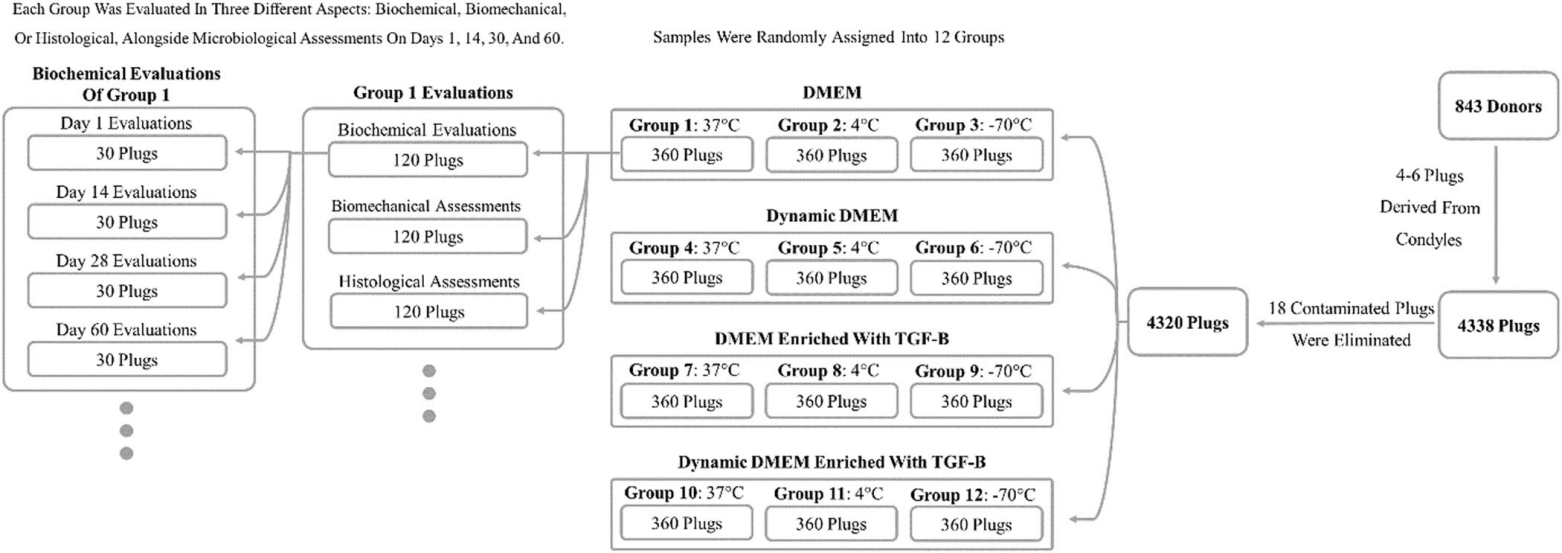
Overview of the experimental setup and evaluation strategy. Osteochondral plugs were divided into 12 groups, each with different combinations of storage conditions (temperature, culture media and dynamic/static culture conditions). Each group was assessed for biochemical, biomechanical, histological, and microbiological parameters at four time points. DMEM, Dulbecco's modified Eagle's medium; TGF‐β, transforming growth factor‐beta.

Subsequently, harvested plugs were randomly assigned to 12 different groups (Table 1), using the block randomization method. These 12 different groups comprised combinations of two culture media (Media 1 or Media 2), two culture conditions (static or dynamic), and three temperatures (−70°C, 4°C and 37°C). Figure 1 illustrates the experimental setup strategy.

**Table 1:** The characteristics of the groups based on different temperatures and storage conditions.

Group	Culture medium	Temperature
1	DMEM	37°C
2	DMEM	4°C
3	DMEM	−70°C
4	Dynamic DMEM	37°C
5	Dynamic DMEM	4°C
6	Dynamic DMEM	−70°C
7	DMEM enriched with TGF‐β	37°C
8	DMEM enriched with TGF‐β	4°C
9	DMEM enriched with TGF‐β	−70°C
10	Dynamic DMEM enriched with TGF‐β	37°C
11	Dynamic DMEM enriched with TGF‐β	4°C
12	Dynamic DMEM enriched with TGF‐β	−70°C

Abbreviations: DMEM, Dulbecco's modified Eagle's medium; TGF‐β, transforming growth factor‐beta.

Media 1 was formulated to deliver fundamental nourishment to the tissue and comprised 20 cc of serum‐free culture medium, namely Dulbecco's modified Eagle's medium (DMEM)/Ham's nutrient mixture F12, supplemented with l‐glutamine, ascorbic acid, penicillin, amphotericin and streptomycin. Media 2 was designed to possess both anti‐inflammatory and chondrogenic properties and was an augmentation of Media 1 with an additional 10 ng/mL of TGF‐β.

For dynamic culture conditions, samples were placed on an orbital shaker (Heidolph Instruments GmbH & Co. KG) daily for 3 h to induce fluid flow around the plugs, simulating synovial fluid movement during knee motion. Additionally, the medium was continuously replaced using an ambulatory infusion pump (Accumate® 1000; Wooyoung Medical). For static culture conditions, the culture medium was refreshed every 48–72 h without inducing any flow in the medium.

Post‐culture, the plugs from each of these experimental groups were subjected to refrigeration at 4°C, incubation at 37°C, or a controlled freezing process at consistent cooling rates following exposure to either glycerol or dimethyl sulfoxide at −70°C (a cryopreservation procedure), based on the temperatures assigned to them as outlined in Table 1.

Control osteochondral plugs (the first plug from each set of four to six plugs derived from the same donor) were analyzed (as outlined in ‘Transfer and preservation conditions’ section) immediately after harvesting (Day 1), without any storage treatment, to serve as a baseline for comparison. These samples were then placed in 10% formalin for preservation.

**Figure 2:**
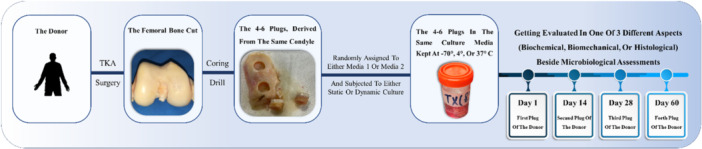
Schematic of the experimental workflow, showing osteochondral plug extraction from donors, random assignment to different media (DMEM or TGF‐β enriched), and incubation conditions (static or dynamic) at various temperatures (−70°C, 4°C or 37°C). Evaluations were conducted on Days 1, 14, 28 and 60 to assess tissue quality through biochemical, biomechanical, histological, and microbiological analyses. DMEM, Dulbecco's modified Eagle's medium; TGF‐β, transforming growth factor‐beta; TKA, total knee arthroplasty.

Subsequently, the plugs were extracted from storage at specific intervals (14, 28 and 60 days after the retrieval of condyles from the donors) and subjected to evaluations by the description outlined in ‘Transfer and preservation conditions’ section (Figure 2 shows experimental workflow). Although no indications of thermal harm to the cartilage resulting from the coring drill were detected, a precautionary measure was taken by discarding the outermost 1‐mm region of cartilage before analysis. This step was taken to eliminate any potential damage that might have occurred during the harvesting process.

### Comprehensive evaluations

Four different types of evaluations were conducted in this study: biochemical, biomechanical, histological, and microbiological assessments. The biochemical evaluations included chondrocyte viability, proteoglycan content and glycosaminoglycan (GAG) content assessments.

To minimize bias, evaluation results from each plug on each day were compared with those from a control plug taken from the same donor on Day 1. For example, in the biomechanical assessment within Group 1, which involved 120 plugs from 30 patients, results from the second plug of the donor ‘A’ on Day 14 were compared with the first (control) plug of the same donor ‘A’ on Day 1 (Figure 2). Additionally, each plug was assigned to a single assessment to maintain data integrity and prevent cross‐testing effects.

#### Chondrocyte viability

At each time point, the full‐thickness articular cartilage was meticulously detached from the subchondral bone and subsequently sliced into coronal sections, approximately 0.5 mm thick, utilizing a scalpel. The cellular specimens were subjected to a 15‐minute exposure of acridine orange solution (Sigma‐A9231) at ambient temperature. Subsequently, the samples were washed three times with phosphate‐buffered saline (PBS; Sigma‐P4417). After washing, propidium iodide (PI; Sigma‐P4864) was added, followed by another series of three PBS rinses.

Subsequently, the specimens were examined using a confocal laser microscope, and images were captured at three randomly selected locations within distinct regions of the cartilage: the superficial region (<0.7 mm deep), the middle region (0.7–1.4 mm deep) and the deep region (>1.4 mm deep). For each plug, a total of nine images were obtained and then averaged to derive a single data point. Live and dead cells were quantified to calculate the percentage of cell viability [26].

#### GAG content

A GAGs ELISA kit (Catalogue no. MBS730728; MyBioSource, Inc.) using a competitive enzyme immunoassay was applied, incorporating a polyclonal anti‐GAGs antibody alongside a GAGs‐horseradish peroxidase (HRP) conjugate. Samples and buffer were co‐incubated with the GAGs‐HRP conjugate on a pre‐coated plate for 1 h. After incubation, the wells were decanted and washed five times. The wells were then incubated with a substrate for the HRP enzyme. The product of the enzyme‐substrate reaction forms a blue‐coloured complex. Finally, a stop solution was added to stop the reaction, which would then turn the solution yellow. Colour intensity, read at 450 nm on a microplate reader, was inversely proportional to the GAG concentration due to the competition between sample GAGs and GAG‐HRP for binding sites on the antibody. A standard curve, plotting optical density (OD) against known GAG concentrations, allowed interpolation of sample GAG concentrations from their absorbance values.

#### Proteoglycan content

The microplate kit (Catalogue no. MBS2706049; MyBioSource Inc.) used in this assay was pre‐coated with an antibody specific to proteoglycan 4. Standards and samples were added to the wells and incubated for 1 h at 37°C. Subsequently, the liquid in wells was removed, and a biotin‐conjugated antibody specific to proteoglycan 4 was added. After incubation (for 1 h at 37°C). Next, liquids in wells were aspirated and washed three times. In the following, Avidin conjugated to HRP was added to each well and incubated for 30 min at 37°C, followed by aspiration of the solution and wash step. After adding tetramethylbenzidine substrate solution, a colour change occurred only in wells containing proteoglycan 4 bound to both the biotin‐conjugated antibody and enzyme‐conjugated avidin. After incubation for 10–20 min at 37°C, the enzyme‐substrate reaction was terminated by adding sulphuric acid, and the colour change was measured spectrophotometrically at a wavelength of 450 ± 10 nm. Subsequently, the proteoglycan 4 concentration in the samples was determined by comparing the OD value of the samples to the standard curve.

#### Uniaxial compression relaxation test

Uniaxial compression relaxation testing is a mechanical test widely used to assess the viscoelastic properties of materials [21], including biological tissues like cartilage. The test involves applying a compressive force to the sample along a single axis and measuring the resulting deformation and stress response. The stress response of the sample is calculated by dividing the applied force by the cross‐sectional area of the sample. During the compression phase of the test, the sample is compressed at a constant rate until a predetermined strain is achieved. Once the strain is reached, the force is held constant for a specified time. This allows the sample to relax and recover some of its original shape, which is then measured and used to calculate the mechanical properties of the material.

In the present study, compression relaxation experiments were performed on each sample, using a strain amplitude of 10% and a duration of 20 s. The tests were carried out using the uniaxial material testing device with a 500 N load cell. The Stress was calculated by dividing the applied force by the cross‐sectional area. The strain was calculated by dividing the change in length by the initial length. All experiments were conducted at the same temperature to ensure consistency in the results. Special care was taken to ensure the samples remained moist throughout the experiments. This was important because their water content can affect the viscoelastic properties of biological tissues like cartilage. Moreover, to prevent samples from slipping during the tests, a custom‐designed fixture was used that provided a secure grip on the samples. The fixture was designed to apply the compressive force evenly on the samples and prevent any lateral movement that could affect the results. The samples were also carefully aligned with the loading axis to guarantee accurate measurements of stress and strain.

#### Bacterial surveillance

On the 28th and 60th days, the media underwent routine processing for microbial culture. The media samples were introduced onto MacConkey agar and triple Blood/Chocolate/EMB agar for identification of Gram‐positive, Gram‐negative and *Candida* species. Incubation occurred at 35°C for 48 h.

To identify Gram‐positive organisms, established methods were used, including Gram staining, catalase and coagulase tests, along with the Thermo‐Fisher Sensititre AP‐90 Automated System (Thermo‐Fisher). Similarly, Gram‐negative organisms were identified through standard methodologies and the AP‐80 Gram‐negative Automated System (Thermo‐Fisher). For antimicrobial susceptibility testing, the Thermo‐Fisher Sensititre Broth Microdilution system was employed. All positive samples, along with other samples obtained from the same donor, were excluded from the study.

#### Histological evaluations

Samples were collected on Days 1, 14, 28 and 60 and were fixed in 10% buffered formalin for at least 48 h, decalcified in 10% EDTA, embedded in paraffin, and sectioned. Sections were stained with haematoxylin and eosin to evaluate general tissue architecture and cellular details, and with Toluidine blue to specifically assess proteoglycan distribution within the ECM. Histological evaluations were conducted using the Osteoarthritis Research Society International scoring system, which assesses parameters such as cartilage structure, chondrocyte morphology, proteoglycan content, collagen organization, integrity of the tidemark and subchondral bone alterations. Each parameter contributes to a cumulative score, with a maximum potential pathological score of 60; higher scores indicate more severe histopathological abnormalities. Two board‐certified pathologists, blinded to the treatment groups, independently scored the samples. For each sample, two sections were evaluated, and the mean score was calculated for each pathologist. The average scores from both pathologists were then used for data analysis.

### Sample size

Based on our pilot study, a sample size of 30 condyles per evaluation for each group was considered sufficient, assuming an *α* of 0.05, a power of 0.8 and the results of the stress relaxation test as the primary outcome with a standard deviation of 0.03 for one‐way analysis of variance (ANOVA).

### Statistical analysis

Statistical analysis was conducted using one‐way ANOVA. For percentage data, arcsine transformation was applied prior to analysis. Unless otherwise specified, the data were presented as mean ± standard deviation. All statistical analyses were performed with SPSS for Windows (version 22), and with statistical significance set at *p* < 0.05.

## RESULTS

The study comprised 843 donors (58.0% female and 42.0% male), aged between 51 and 65 years. From the initial collection of 4338 harvested cartilage plugs, 18 samples were excluded due to contamination, which could potentially bias the biochemical, biomechanical and histological analyses. The remaining 4320 plugs were evenly allocated into 12 distinct tissue preservation protocols, with each group containing 360 plugs, as detailed in Table 1 and illustrated in Figure 1.

Each group underwent analysis across three assessment categories (biochemical, biomechanical and histological evaluations), with all media of groups subjected to microbiological surveillance. This resulted in a total of 1440 plugs being analyzed within each category. Baseline assessments were performed on the 1080 control plugs on Day 1, which had no exposure to culture conditions.

### Chondrocyte viability

A one‐way ANOVA was performed to compare the mean viability across 12 groups, revealing a significant difference in viability between groups on Days 28 and 60 of sample storage. Subsequent analyses using one‐way ANOVA and Tukey's HSD post hoc test showed that among different temperatures, samples stored at 37°C had significantly higher viability, with a mean viability of 82.18 ± 3.67% (*p* < 0.001, $\eta_{P}^{2}$ = 0.10) on Day 28 and 65.07 ± 4.26% (*p* < 0.001, $\eta_{P}^{2}$ = 0.06) on Day 60. No significant viability differences were observed between the samples stored at 4°C and −70°C.

Additionally, the effect of different culture media on OCA sample viability was assessed. Results indicated that samples stored in dynamic DMEM enriched with TGF‐β had significantly higher mean viability on Day 28 with a mean viability of 82.14 ± 4.12% (*p* < 0.001, $\eta_{P}^{2}$ = 0.13) and Day 60 with a mean viability of 66.83 ± 3.55% (*p* < 0.001, $\eta_{P}^{2}$ = 0.28), indicating a substantial effect.

**Figure 3:**
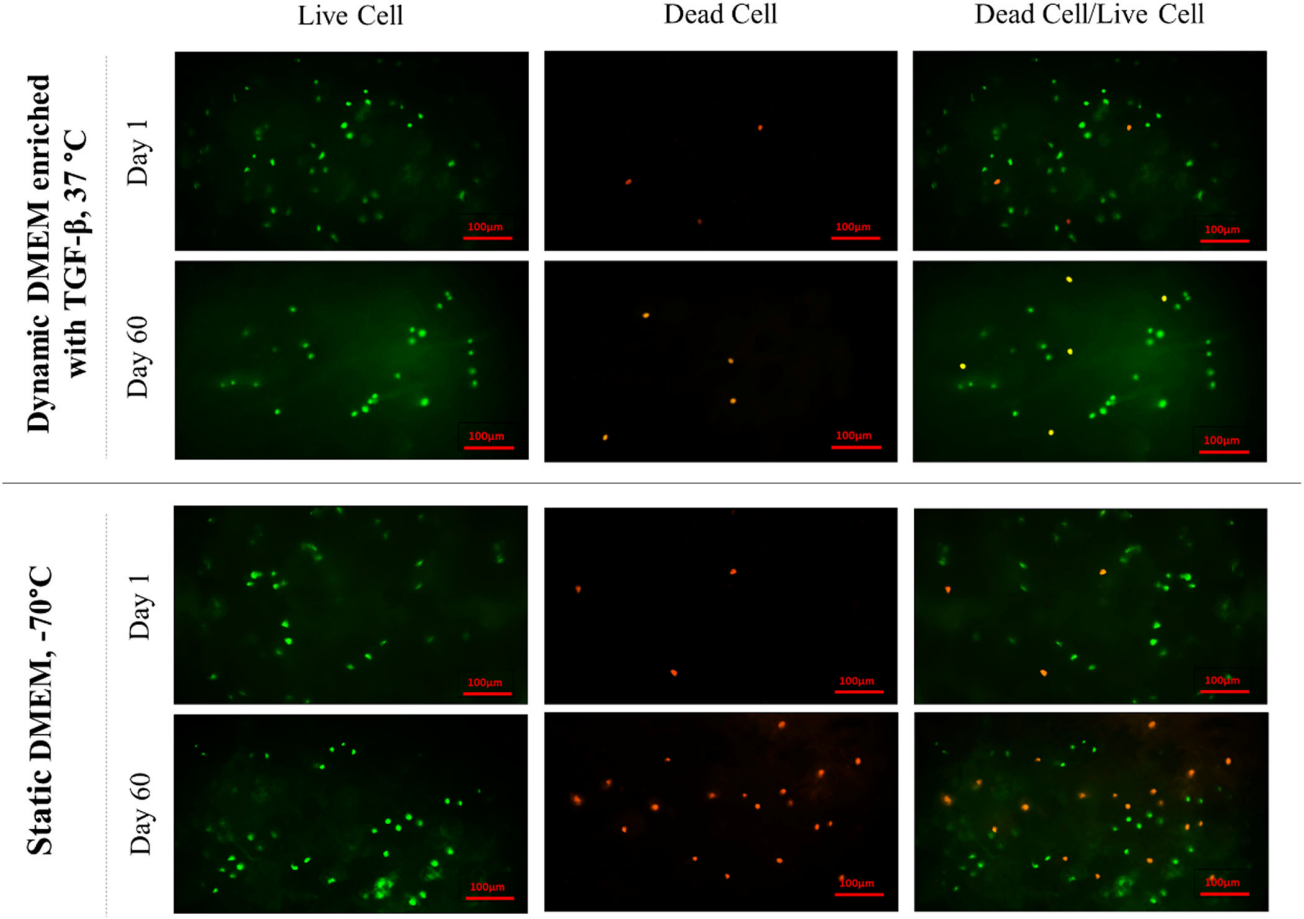
An example of fluorescence microscopic evaluation of chondrocyte viability in two distinct storage conditions: Dynamic DMEM enriched with TGF‐β at 37°C and Static DMEM at −70°C on Days 1 and 60. As shown, the dynamic DMEM culture condition enriched with TGF‐β at 37°C, markedly preserves chondrocyte viability better than the static DMEM culture condition at −70°C. DMEM, Dulbecco's modified Eagle's medium; TGF‐β, transforming growth factor‐beta.

Specifically, samples stored in dynamic DMEM enriched with TGF‐β at 37°C (Group 10) demonstrated significantly a higher viability on Day 28 with a mean viability of 85.4 ± 2.87% (*p* < 0.001, $\eta_{P}^{2}$ = 0.28) and Day 60 with a mean viability of 68.70 ± 3.97% (*p* < 0.001, $\eta_{P}^{2}$ = 0.36). Groups 11, 5 and 8 also exhibited significantly better viability than other groups, after Group 10. Table 2 details the mean viability of the groups on Days 28 and 60. Figure 3 shows an example of cell viability assessment using a fluorescence microscope on Days 1 and 60.

**Table 2:** Mean viability of the groups on Days 28 and 60.

Group	Mean viability on Day 28	Mean viability on Day 60
1	76.97%	60.30%
2	80.00%	61.30%
3	77.67%	59.93%
4	80.53%	64.17%
5	81.60%	65.10%
6	80.07%	62.53%
7	80.47%	61.77%
8	81.70%	65.17%
9	80.43%	61.63%
10	85.40%	68.70%
11	81.93%	65.80%
12	79.10%	66.00%

*Note*: 1–3: DMEM at 37°C, 4°C and −70°C; 4–6: Dynamic DMEM at 37°C, 4°C and −70°C; 7–9: DMEM with TGF‐β at 37°C, 4°C and −70°C; 10–12: Dynamic DMEM with TGF‐β at 37°C, 4°C and −70°C.

Abbreviations: DMEM, Dulbecco's modified Eagle's medium; TGF‐β, transforming growth factor‐beta.

### Proteoglycan synthesis

**Figure 4:**
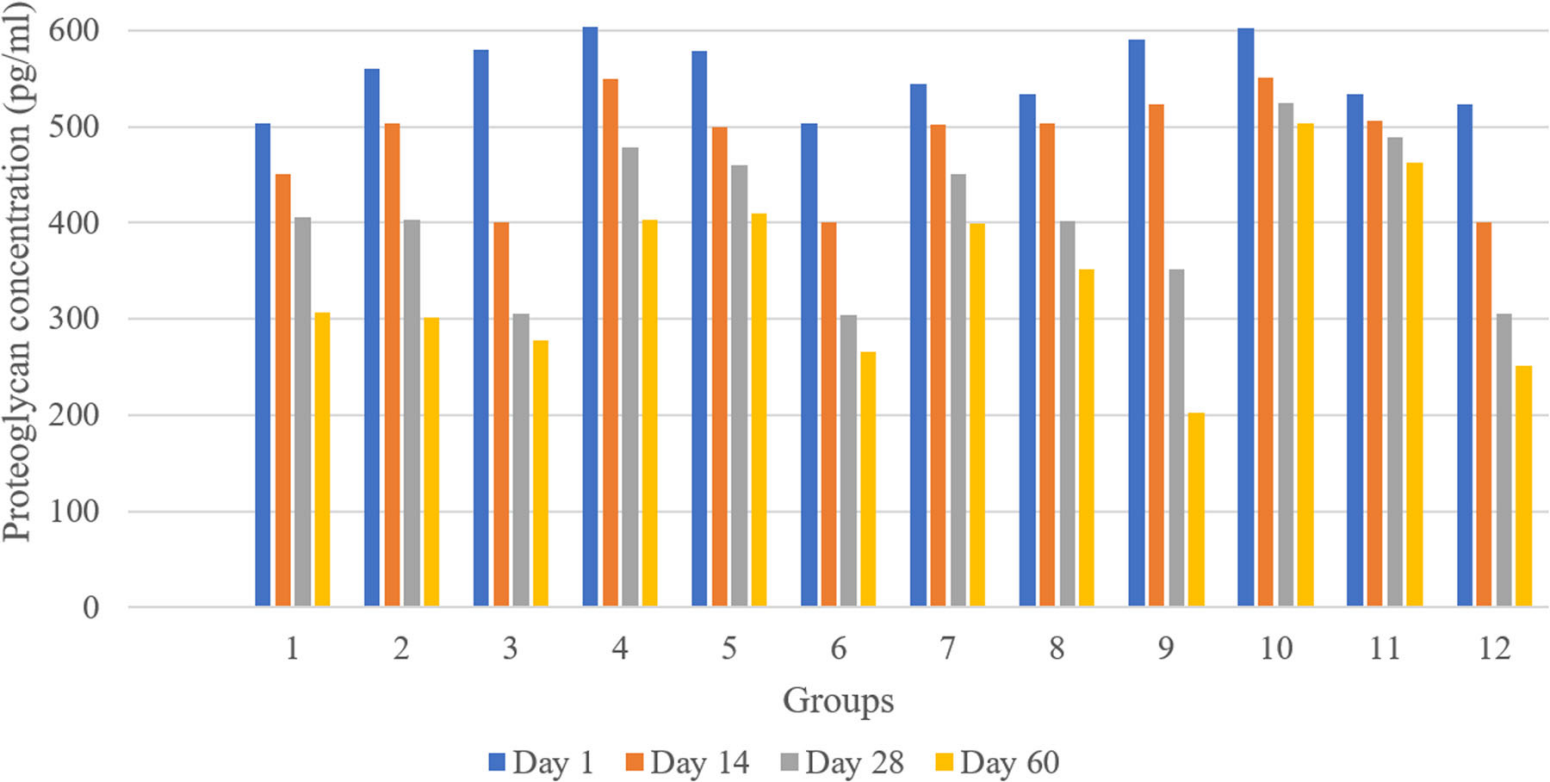
Proteoglycan concentration across groups with various culture media and storage conditions. 1–3: DMEM at 37°C, 4°C and −70°C; 4–6: Dynamic DMEM at 37°C, 4°C and −70°C; 7–9: DMEM with TGF‐β at 37°C, 4°C and −70°C; 10–12: Dynamic DMEM with TGF‐β at 37°C, 4°C and −70°C. DMEM, Dulbecco's modified Eagle's medium; TGF‐β, transforming growth factor‐beta.

To evaluate the significance of the differences in concentration reductions among groups, a one‐way ANOVA was conducted for each time point (Days 1, 14, 28 and 60). Subsequently, post hoc tests were performed to identify specific group pairs with significant differences. The summarized results are presented in Figure 4.

On Days 1 and 14, no significant differences in concentration reductions were observed between any groups (Table 3). However, on Day 28, Group 10 showed significantly higher concentrations compared to Group 3 (*p* = 0.0136), Group 6 (*p* = 0.0131) and Group 9 (*p* = 0.0359).

**Table 3:** ANOVA results comparing concentration reductions of proteoglycan content across all groups on Days 1 and 14.

Day	*F*‐statistic	*p* value
1	0.9076	0.5395
14	1.7615	0.1805
28	3.7847	0.0153
60	4.9913	0.0043

Abbreviation: ANOVA, analysis of variance.

On Day 60, Group 10 maintained significantly higher concentrations compared to Group 1 (*p* = 0.0334), Group 2 (*p* = 0.0247), Group 3 (*p* = 0.0131), Group 6 (*p* = 0.0131) and Group 9 (*p* = 0.0098). Additionally, Group 11 showed significantly higher concentrations than Group 9 (*p* = 0.0015).

Overall, Groups 10 and 11 appeared to maintain higher concentrations over time compared to other groups, particularly by Day 60. In contrast, Groups 3, 6 and 9 showed the most substantial reductions in concentration by Day 60.

### GAG content

Based on the ANOVA results, no statistically significant differences were observed in GAG concentration between the 12 storage protocols at any time point (Days 1, 14, 28 or 60). This finding suggests that the reduction in GAG concentration over time was similar across all storage environments. Although a general trend of decreasing GAG concentration over time was observed across all groups, the differences between groups at each time point were not statistically significant.

The average reduction in GAG concentration from Days 1 to 60 across all groups was approximately 48.7%. The highest reduction was observed in Group 9, with a 47.8% decrease, while the lowest reduction was seen in Group 10, with a 40.4% decrease.

**Figure 5:**
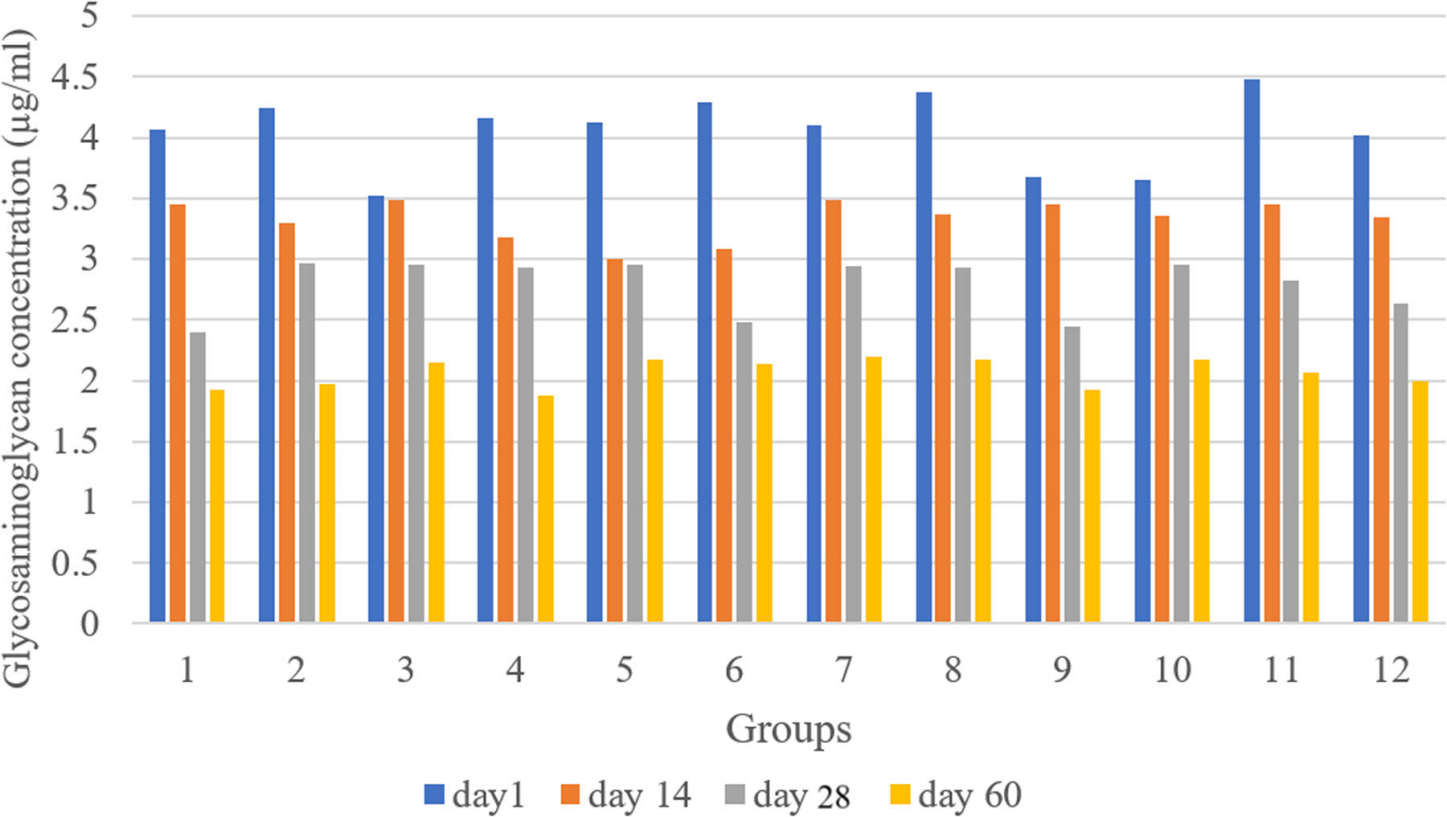
GAG concentration in groups with various culture media and storage conditions. 1–3: DMEM at 37°C, 4°C and −70°C; 4–6: Dynamic DMEM at 37°C, 4°C and −70°C; 7–9: DMEM with TGF‐β at 37°C, 4°C and −70°C; 10–12: Dynamic DMEM with TGF‐β at 37°C, 4°C and −70°C. DMEM, Dulbecco's modified Eagle's medium; GAG, glycosaminoglycan; TGF‐β, transforming growth factor‐beta.

Despite these variations, the statistical analysis indicated that none of the storage environments preserved GAG concentration significantly better or worse than others (Figure 5).

### Biomechanical properties

**Figure 6:**
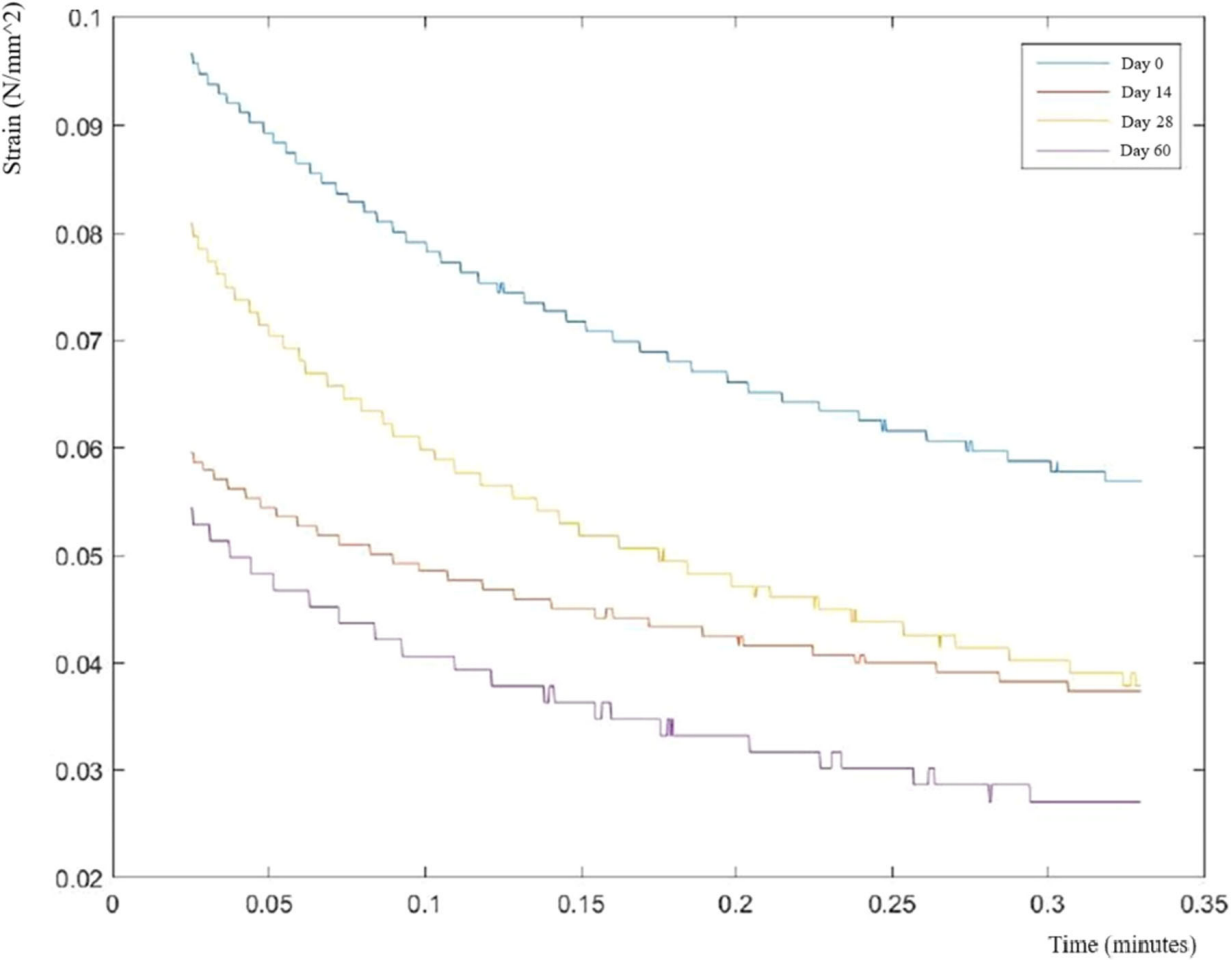
Stress relaxation response over time for a representative group, showing a decrease in peak stress at various evaluation points (Days 0, 14, 28 and 60). Similar decreasing trends were observed across other groups.

A one‐way ANOVA was conducted to analyze the changes in the stress relaxation test results in the groups, revealing a significant difference in the biomechanical status of the groups on Day 60 of storage. The overall mean reduction in resistance to stress was 0.06142 N/mm^2^. Interestingly, Group 12 exhibited a significantly lower mean reduction in resistance to stress (0.03357 N/mm², *p* < 0.001) compared to other groups. The post hoc tests indicated that Groups 11, 6 and 10 also had lower mean reductions following Group 12 (Figure 6).

The one‐way ANOVA results indicated a significant effect of storage temperatures on the results of the mean reductions (*p* < 0.001). The post hoc test revealed that samples stored at −70°C had the lowest mean reduction in resistance (0.04747 N/mm^2^), which was significantly less than those stored at 4°C (0.06371 N/mm^2^), and at 37°C (0.07309 N/mm^2^).

Furthermore, the analysis showed a significant effect of culture media type on the results (*p* < 0.001). The mean reduction in resistance to stress for each culture medium was as follows: dynamic DMEM enriched with TGF‐β = 0.03959, dynamic DMEM = 0.06110 N/mm^2^, DMEM enriched with TGF‐β = 0.06836 N/mm^2^ and DMEM = 0.07665 N/mm^2^. As a result, samples in dynamic DMEM enriched with TGF‐β exhibited the lowest reduction in resistance, significantly outperforming other media.

### Histological evaluations

All specimens exhibited degenerative changes during processing, including chondrocyte swelling, nuclear enlargement and cell hypertrophy accompanied by varying degrees of matrix degradation.

**Figure 7:**
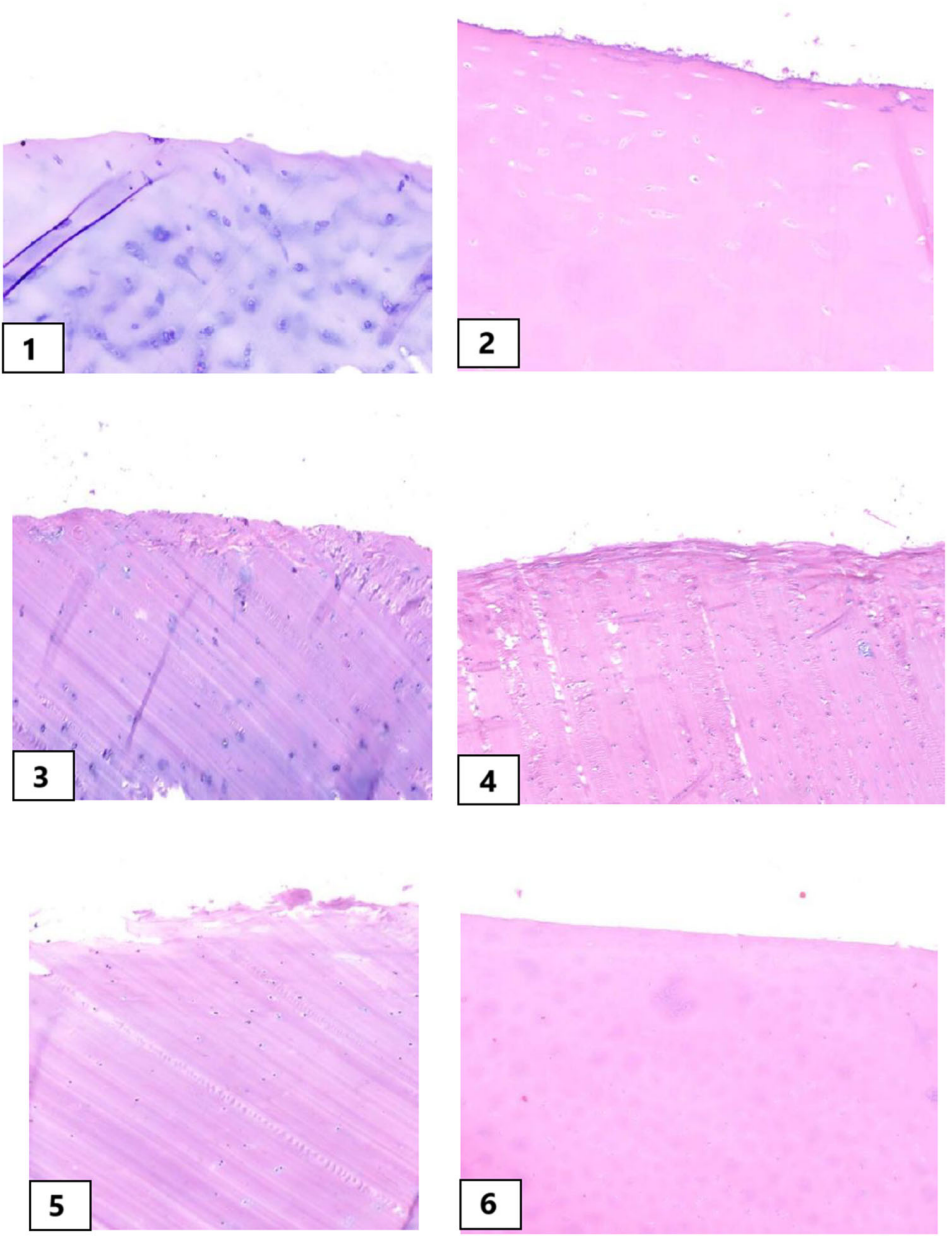
Histological evaluation of osteochondral plugs preserved in DMEM culture media. All specimens exhibited degenerative changes alongside matrix degradation. While no significant qualitative differences were observed between preservatives, samples at −70°C displayed more prominent nuclear apoptosis. 1–2: Static DMEM culture at 37°C on Days 0 and 60; Static DMEM culture at 4°C on Days 0 and 60; 5–6: Static DMEM culture at −70°C on Days 0 and 60. DMEM, Dulbecco's modified Eagle's medium.

No significant qualitative differences were observed between the various preservatives; however, the 4°C and TGF‐β groups showed slightly better results (*p* > 0.05). Regarding various temperatures, samples at −70°C showed more prominent nuclear apoptosis. Figure 7 presents examples of histologic evaluations.

### Bacterial surveillance

Overall, 18 samples were contaminated in this study: 6 samples (42%) in Group 7, 5 samples (28%) in Group 1, 4 samples (16%) in Group 8 and 3 samples (14%) in Group 4. Fourteen cases were contaminated on Day 28, and four cases on Day 60. Specifically, eight cases were contaminated with *Staphylococcus aureus*, four cases were contaminated with *Sphingomonas paucimobilis* and six cases were contaminated with *Bacillus* and *Proteus* species. All contaminated samples were eliminated from the biochemical, biomechanical and histological evaluations. Most contamination occurred in samples stored at 37°C. However, the differences in contamination rates and bacterial culture results across groups were not statistically significant (*p* > 0.05).

## DISCUSSION

The study evaluated the quality of macroscopically healthy cartilage (from patients undergoing TKA) after storage, examining how different storage durations, temperatures, static and dynamic conditions, and preservation solutions affect cartilage quality. The findings provide valuable insights into optimizing OCA transplantation. Among the protocols examined, storage of OCAs in Media 2 at 37°C subjected to dynamic culture conditions resulted in the best maintenance of chondrocyte viability, ECM biochemical composition, and biomechanical properties compared to the other storage methods. The hypothesis was confirmed that storage at physiologic temperature (37°C) in TGF‐β supplemented media enhances viability compared to other storage conditions. Additionally, OCAs stored in media subjected to dynamic culture conditions exhibited improved biomechanical characteristics compared to OCAs stored in static media.

In a systematic review, Tabba et al. found that the effects of storage temperatures on OCA health varied across studies, with inconsistent outcomes reported for storage at 37°C or room temperature (21–25°C). Approximately half of the studies evaluating OCA storage at 37°C showed improvement, while the other half indicated deterioration. Similar inconsistencies were also noted at room temperature. These discrepancies likely reflect differences in experimental design, tissue types, media and outcome measures [34].

Previous studies have suggested that OCA success is a function of the viability of the graft chondrocytes, which diminishes over storage time [24], and storage at physiological temperatures results in favourable chondrocyte viability compared with 4°C [13, 27]. Pallante et al. [27] demonstrated that caprine chondrocyte viability is maintained for up to 28 days at 37°C (approximately 80% at the cartilage surface, 65% in the superficial zone and 70% in the middle zone), while 4°C incubation demonstrated a 30% decrease in viability (approximately 45%, 20% and 35%, respectively). In canine cadaver studies, Garrity et al. [13] reported a mean 28‐day tissue viability of 39.73% in samples maintained at 4°C compared with 76.40% viability for OCAs stored at 37°C and Day 0 control of 77.23% viability. OCAs viability was maintained at fresh levels through 56 days of storage at 37°C (69.55%). The results in the present study align with those reported in previous studies. Here, chondrocyte viability in OCAs was significantly higher on Days 28 and 60 at 37°C compared to 4°C.

In addition, the present study demonstrated that OCAs stored at −70°C, despite the addition of cryoprotective agents, led to a significant decrease in viability compared to the control group. This result aligns with previous findings on freezing intact articular cartilage, which results in either complete or near‐complete loss of chondrocyte viability. Denbeigh et al. [11] compared storage of OCAs at two commonly proposed storage temperatures, room temperature (~22–25°C) and physiologic temperature (37°C), noting that these conditions had not been previously compared side by side for human tissues, and this comparison is important for the development and future standardization of OCA storage protocols. The results of their study demonstrated higher viability in the physiologic temperature storage group than in the room temperature storage group.

The effects of different storage times and media recipes on cellular viability have been widely studied in the past and summarized by De Caro et al. [10] and Wright et al. [41]. In canine cadaver studies, Garrity et al. [13] compared chondrocyte viability, as well as biochemical and biomechanical properties of OCAs stored in Media 1 (similar to the current standard) or Media 2 (an anti‐inflammatory and chondrogenic media containing dexamethasone and TGF‐β) at 4°C or 37°C for up to 56 days. Storage in media containing dexamethasone and TGF‐β demonstrated significantly lower levels of chondrocyte viability and GAG content than standard media. Contrary to the results of Garrity's study, the present study showed that adding TGF‐β significantly enhanced the chondrocyte viability, proteoglycan synthesis and GAG content. However, TGF‐β did not improve the biomechanical properties of osteochondral allografts. Additionally, the current research compared dynamic and static cultivation conditions, revealing that dynamic culture conditions significantly improved the biomechanical properties of osteochondral allografts as well as proteoglycan synthesis. However, culture conditions did not affect chondrocyte viability. While the impact of medium compounds on cell viability was demonstrated in this study, storage temperature remained the most influential factor, aligning with previous reports [27, 35].

To date, storage at 37°C has not become standard practice due to existing tissue banking regulations and concerns related to cost or microbial contamination at temperatures above 4°C. A recent study by Stoker et al. [33] indicated that storage at room temperature is safe, because all tissue and media samples passed sterility tests, showing no microbial growth. Garrity et al. [13] also demonstrated that microbial cultures of the media collected at the end of storage at 37°C caused no increase in bacterial contamination. Nevertheless, in the current study, microbiological investigations on day 60 showed that storage at 37°C was associated with an increase in the incidence of bacterial contamination compared to 4°C. This was likely due to more favourable growth conditions at 37°C, though the result was not statistically significant, suggesting that these variations may be due to random factors rather than a systematic effect related to the storage conditions or group assignment. Moreover, it is still possible that some of these samples became contaminated during the handling procedure. All contaminated samples, along with other plugs obtained from that donor, were excluded from the study. Replacement samples from other donors were used to maintain data integrity and prevent potential biases that could affect the results. This approach minimized errors and ensured that the analysis of cell viability, as well as biomechanical and biochemical properties, remained reliable and reflective of uncontaminated conditions.

The storage at 4°C was originally desired and recommended due to the theoretical reduction in microbiological viability and growth. However, as demonstrated before, storage below physiological temperatures also negatively impacts cartilage viability. With increasing evidence supporting aseptic storage at 37°C, the justification for cold storage due to microbiological concerns is becoming less compelling.

Despite advancements in preservation techniques aimed at extending storage duration, significant limitations persist due to the narrow window between extraction and transplantation. Additionally, the scarcity of healthy cadaveric cartilage candidates exacerbates these challenges. Moreover, there is a significant inter‐specimen and intra‐study variability in chondrocyte viability of OCAs at the time of implantation [19]. This variability is attributed to differences in harvest timing, technique, location, and the initial condition of the cartilage, all of which can significantly influence the graft's quality and performance [37]. To address these issues, we propose the establishment of a living donor cartilage programme. This approach could enhance graft availability, provide grafts with high cell viability, enable donor prescreening for safety, facilitate more flexible surgical scheduling, and potentially offer a cost‐effective solution. The successful transplantation of OCAs from living donors has been previously reported [15].

There are limitations in the present study. Primarily, the study was conducted only under in‐vitro conditions, without in vivo validation. Although we assessed cell viability as well as histological, biochemical and biomechanical indicators of tissue quality, none of these provide a direct assessment of in vivo function or clinical success. To address this limitation, further translational research involving animal models is necessary. Additionally, previous studies showed a regional variation within the cartilage in response to storage, which has not been investigated in this study. Second, although we aimed to use areas with shiny, macroscopically healthy cartilage, the samples in this study, derived from osteoarthritic joints affected by degenerative changes and enzymatic activity, and may have been more fragile and sensitive compared to fresh, healthy cartilage from cadaveric sources. Consequently, using fresh cadaveric samples might have yielded better outcomes. However, obtaining samples from TKA candidates, made it possible for us to conduct this examination with a significantly larger sample size, allowing for a robust comparison across 12 storage protocols with 30 samples per test and culture condition for each time point, which is an achievement unprecedented in similar studies. Our study uniquely integrates various temperatures, media compositions, and dynamic/static conditions to provide a comprehensive assessment of osteochondral tissue preservation, unlike previous studies that typically focused on single variables. Additionally, dynamic culture conditions were employed, providing valuable insights into how fluid flow and continuous media replacement impact tissue preservation, an aspect seldom explored in human samples. Moreover, TGF‐β was employed here to optimize storage conditions, a novel application in osteochondral preservation research.

## CONCLUSION

The results indicated optimal storage conditions for femoral condyle OCAs involve using Media 2 at moderate temperatures (37°C and 4°C). These conditions support better chondrocyte viability and proteoglycan synthesis. However, challenges persist in maintaining GAG content and biomechanical properties over time, with viscoelasticity observed, particularly in dynamic culture conditions. The histological evaluation revealed degenerative changes and certain storage conditions increased the risk of bacterial contamination.

Storing OCAs at 37°C in serum‐free chemically defined media supplemented with TGF‐β subjected to dynamic culture conditions has important potential advantages, compared with the current standard‐of‐care 4°C protocols used by tissue banks. Prior research has shown that storage at 37°C is superior to 4°C at 28 days, and the present study supports those findings while demonstrating that OCAs can be stored for up to 60 days with maintained chondrocyte viability and tissue quality. If this novel storage protocol becomes validated with respect to in vivo safety and efficacy, it could effectively quadruple the clinical window for OCA grafting from 14 to 60 days. This longer storage period would enhance the availability of high‐quality OCAs for patients, reduce costs, and decrease the number of discarded grafts.

This study provides valuable contributions to the field of joint resurfacing techniques and may pave the way for more effective osteochondral allograft transplantation, benefiting patients with cartilage defects and osteoarthritis.

## AUTHOR CONTRIBUTIONS

*Conceptualization, project administration, supervision, funding acquisition, and methodology*: Mohammad Hossein Nabian, Fardis Vosoughi and Leila Oryadi Zanjani. *Writing—original draft, data curation, formal analysis and investigation*: Ramin Shayan‐Moghadam. *Formal analysis and investigation*: Hossein Nematian. *Drafting of the article and data collection*: Arash Sherafatvaziri and Alireza Mirzamohamadi. *Analysis and interpretation of the data*: Hiva Saffar, Mahdi Shafieian. All authors read and approved the final manuscript.

## CONFLICT OF INTEREST STATEMENT

The authors declare no conflicts of interest.

## ETHICS STATEMENT

The study was performed in accordance with the ethical standards laid down in the 1964 Declaration of Helsinki. The institutional research ethics committee approved the study protocol on 2022‐02‐12 with the approval code IR.TUMS.MEDICINE.REC.1400.1338.

## Data Availability

The data that support the findings of this study are available from the corresponding author upon reasonable request.
